# Dose–Response of Intracameral Bimatoprost Sustained-Release Implant and Topical Bimatoprost in Lowering Intraocular Pressure

**DOI:** 10.1089/jop.2018.0095

**Published:** 2019-04-06

**Authors:** Susan S. Lee, Mohammed Dibas, Alexandra Almazan, Michael R. Robinson

**Affiliations:** Allergan plc, Irvine, California.

**Keywords:** bimatoprost, drug delivery, eye drop, intracameral, intraocular pressure, prostaglandin analog

## Abstract

***Purpose:*** To compare the dose–response profiles of bimatoprost sustained-release implant (Bimatoprost SR) and topical bimatoprost in lowering intraocular pressure (IOP) in normotensive beagle dogs.

***Methods:*** In 1 study, topical bimatoprost 0.001%, 0.01%, or 0.1% was administered twice daily in the study eye for 5 days. IOP was measured at baseline and up to hour 6 each day. Other studies evaluated the IOP response to a single administration of Bimatoprost SR at dose strengths ranging from 8 to 120 μg. IOP was measured before implant administration and during 3 months of follow-up; IOP in response to topical bimatoprost 0.03% was measured prestudy as an internal control.

***Results:*** Mean percentage decrease in IOP from baseline at hour 6 (peak effect) across study days was 15.7%, 36.1%, and 24.8% (2.8, 7.0, and 4.0 mmHg) in animals treated with topical bimatoprost 0.001%, 0.01%, and 0.1%, respectively. After Bimatoprost SR administration, mean percentage decrease in IOP from baseline across 3 months consistently increased with increasing dose strength and was 38.7% (7.2 mmHg) with Bimatoprost SR 120 μg. Mean percentage IOP decrease with topical bimatoprost 0.03% was 27.6% (5.9 mmHg).

***Conclusions:*** Topical bimatoprost demonstrated a U-shaped dose–response curve; increasing the bimatoprost concentration to 0.1% resulted in reduced IOP-lowering efficacy. In contrast, the dose–response curve for Bimatoprost SR showed consistently greater IOP lowering as the dose strength increased, with the dose strength producing maximum IOP lowering not yet determined. At 60- and 120-μg dose strengths, Bimatoprost SR produced greater IOP reductions than were achieved with topical dosing.

## Introduction

Topical prostaglandin analogs (PGAs) are commonly used as first-line therapy in glaucoma and ocular hypertension.^1,2^ These medications (eg, latanoprost, tafluprost, travoprost, and the prostamide bimatoprost) are well tolerated and reduce intraocular pressure (IOP) more effectively than other classes of topical IOP-lowering medications.^3,4^

There is a ceiling effect in the IOP lowering produced by the topical PGAs in humans, that is, a leveling off of the effect on IOP despite an increase in drug exposure. Beyond a certain point, increasing drug exposure, either by increasing the concentration of PGA administered or by increasing the dosing frequency, does not increase IOP-lowering efficacy, and in fact, can result in a decrease in IOP lowering and a U-shaped dose–response curve. In the phase 3 clinical trials of bimatoprost 0.03% in patients with glaucoma and ocular hypertension, twice-daily dosing of bimatoprost was significantly less effective than once-daily dosing in lowering IOP at the 10 am time point at all follow-up visits through 6 months.^5^ At month 6, the 10 am mean reduction in IOP from baseline was 8.1 mmHg (32.8%) with bimatoprost QD versus 6.3 mmHg (25.7%) with bimatoprost BID (*P* < 0.001).^5^ However, once-daily and twice-daily dosing of bimatoprost provide similar IOP reductions when the concentration of bimatoprost is lowered to 0.01% and the preservative concentration is varied.^6^ Latanoprost 0.005% or 0.006% also reduced IOP more effectively when dosed once daily than when dosed twice daily in clinical trials of latanoprost in normal subjects^7^ and individuals with ocular hypertension.^8^ Other studies in patients with glaucoma or ocular hypertension have shown that increasing the concentration of latanoprost to 0.0115% or 0.0125% either produces no additional benefit or results in a loss in IOP-lowering efficacy.^9,10^

Bimatoprost sustained-release implant (Bimatoprost SR; Allergan plc, Dublin, Ireland) is a biodegradable implant in development for the treatment of open-angle glaucoma and ocular hypertension.^11^ The implant is solid and rod shaped; it consists of bimatoprost within the biodegradable NOVADUR (Allergan plc) platform for drug delivery, which is constructed from synthetic aliphatic polyesters.^12^ For Bimatoprost SR, the NOVADUR platform was modified to provide nonpulsatile, steady-state release of bimatoprost (ie, zero-order kinetics).^11^ The implant is placed intracamerally and was designed to release bimatoprost slowly to lower IOP for 4–6 months. Interim results of a dose-ranging phase 1/2 study of Bimatoprost SR containing 6, 10, 15, or 20 μg of bimatoprost have been reported and showed that each dose strength effectively lowered IOP in patients with open-angle glaucoma.^11^ The IOP lowering observed was dose dependent for at least the first 12 weeks after implant administration, and mean overall IOP reductions from baseline through week 16 were 7.2, 7.4, 8.1, and 9.5 mmHg with the 6-, 10-, 15-, and 20-μg dose strengths of Bimatoprost SR, respectively.^11^

Preclinical studies have evaluated the IOP lowering produced by Bimatoprost SR and topical bimatoprost in normotensive beagle dogs. The primary objective of the present analysis was to determine whether the IOP-lowering efficacy of Bimatoprost SR is limited or reduced at high-dose strengths of the implant, similar to the dose–response observed with topical administration of PGAs.

## Methods

### Animals

Normotensive beagle dogs of both sexes were used in the studies. The animals were trained before initiating the studies to allow IOP determination without sedation or general anesthesia. The research adhered to the Association for Research in Vision and Ophthalmology (ARVO) statement for the Use of Animals in Ophthalmic and Vision Research and was approved by Allergan's Animal Care and Use Committee.

### Topical bimatoprost studies

A study conducted in 1993 evaluated the IOP-lowering efficacy of topical PGAs at varying concentrations in normotensive beagle dogs (3–9 months of age, 10–15 kg). Each formulation was tested in a group of 7–8 animals. When multiple formulations were tested in the same animal, there was a washout period of at least 1 week between treatments. The results for animals treated with topical bimatoprost are reported here. Topical bimatoprost at 1 of 3 concentrations (0.001%, 0.01%, or 0.1%) was dosed twice daily for 5 days. The bimatoprost formulations were prepared using bimatoprost, sterile water, and inactive ingredients (polysorbate 80 and Tris as a buffering agent). A treatment period of 5 days was selected because a minimum treatment period of 3 days is required for dogs to reach a steady-state IOP reduction with topical bimatoprost; a washout of 1 week is adequate for all dogs to return to their baseline IOPs. Seventeen animals were treated topically with the 0.001%, 0.01%, or 0.1% concentration of bimatoprost; 3 animals were treated topically with bimatoprost 0.1% in the right eye after washout of bimatoprost 0.01% in the left eye.

The study was performed at TSI Redfield Laboratories (Redfield, AR), and the data were analyzed at Allergan. IOP was measured in both eyes using a Digilab Modular One Pneuma Tonometer ([pneumatonometer]; Bio-Rad, Cambridge, MA) at hours 0, 2, 4, and 6 on each study day. Topical proparacaine 0.1% was used for corneal anesthesia during the tonometry. A 20-μL drop of bimatoprost was administered to 1 eye (the study eye) and a 20-μL drop of the vehicle was administered to the fellow eye on each day after the hour 0 and hour 6 IOP measurements.

For each concentration of bimatoprost tested (0.001%, 0.01%, and 0.1%), the hour 6 time point following the morning dose is the peak response in dogs, and the hour 6 mean percentage reduction in IOP from baseline IOP (measured in study eyes at hour 0 on day 1) was calculated for each study day and averaged across all study days.

### Bimatoprost implant studies

Five studies conducted in 2009 in drug-naive, normotensive beagle dogs (9 months to 3 years of age, 8–14 kg) evaluated the IOP-lowering efficacy of a single administration of Bimatoprost SR. The studies were conducted at Allergan, and the animals were housed in an Allergan vivarium or at LA BioMed (Harbor-UCLA Medical Center, Torrance, CA). A total of 50 dogs were used for these studies, and Bimatoprost SR was evaluated at dose strengths ranging from 8 to 120 μg of bimatoprost. In each study, Bimatoprost SR or a placebo implant was administered intracamerally in 1 eye, and the fellow eye was either untreated or received an intracameral placebo implant. The implant size was varied to achieve different dose strengths of Bimatoprost SR, with the concentration of bimatoprost in the polymer matrix remaining constant. A single implant was administered to achieve Bimatoprost SR dose strengths ranging from 8 to 60 μg, whereas two 60-μg implants were administered to achieve the 120-μg dose strength. Details of the individual studies, including the study treatments administered, are summarized in Table 1. A sentinel dog was administered a single implant with a 270-μg dose strength of Bimatoprost SR and demonstrated a very large decrease in IOP (ie, 61% reduction from baseline). The decision was made to not administer this high dose strength to another animal because of the large drop in IOP.

**Table 1.** Studies Evaluating Bimatoprost Sustained-Release Implant in Beagle Dogs

*Study No.*	*Treatment group*	n	*Right eye treatment*	*Left eye treatment*	*Total follow-up (months)*	*Time points for IOP measurement in first 3 months (months)*
1 (*n* = 2)					10	0, 0.25, 0.5, 0.75, 1, 1.25, 1.5, 2, 2.5
	Bimatoprost SR 60 μg	1	Bimatoprost SR 60 μg	None		
	Placebo	1	Placebo	None		
2 (*n* = 6)					9	0, 0.25, 0.5, 0.75, 1, 1.5, 3
	Bimatoprost SR 60 μg	3	Bimatoprost SR 60 μg	Placebo		
	Bimatoprost SR 120 μg	3	Bimatoprost SR 120 μg^a^	Placebo		
3 (*n* = 8)					8	0, 0.25, 0.5, 0.75, 1, 1.5, 2, 3
	Bimatoprost SR 30 μg	4	Bimatoprost SR 30 μg	None		
	Placebo	4	Placebo	None		
4 (*n* = 9)					7	0, 0.25, 0.5, 0.75, 1, 1.5, 2, 3
	Bimatoprost SR 8 μg	3	None	Bimatoprost SR 8 μg		
	Bimatoprost SR 12 μg	3	None	Bimatoprost SR 12 μg		
	Bimatoprost SR 22 μg	3	None	Bimatoprost SR 22 μg		
5 (*n* = 25)					4	0, 0.25, 0.5, 0.75, 1, 1.5, 2, 3
	Bimatoprost SR 8 μg	5	Bimatoprost SR 8 μg	None		
	Bimatoprost SR 12 μg	5	Bimatoprost SR 12 μg	None		
	Bimatoprost SR 22 μg	5	Bimatoprost SR 22 μg	None		
	Bimatoprost SR 30 μg	5	Bimatoprost SR 30 μg	None		
	Placebo	5	Placebo	None		

^a^ Two 60-μg implants were administered to achieve the 120-μg dose strength. All other treatments consisted of a single implant.

Bimatoprost SR, bimatoprost sustained-release implant; IOP, intraocular pressure.

On day 1 (treatment day), dogs were administered atropine (0.022 mg/kg, intramuscular), then anesthetized with an intravenous cocktail of 6.25 mg/kg ketamine, 0.625 mg/kg xylazine, and 0.125 mg/kg acepromazine, and placed on their side. The lid and periocular area were prepped with 5% povidone/iodine, and the eyelids were retracted with an eye speculum. After 3 min, the povidone/iodine was washed out with balanced salt solution, the 25-gauge needle of the Bimatoprost SR applicator was inserted through the clear cornea in the superior temporal quadrant with the aid of a surgical microscope, and the implant was released into the anterior chamber. Following release, the implant sinks to the inferior angle, where it resides. A triple antibiotic ophthalmic ointment (bacitracin, neomycin, and polymyxin B; Bausch & Lomb, Bridgewater, NJ) was applied in the cul-de-sac after the procedure.

Follow-up in the studies ranged from 4 to 10 months. IOP readings were taken in the morning (between 8 and 10 am) without sedation using a Mentor 30 Classic Pneumatonometer (Mentor O & O, Inc., Norwell, MA). A drop of topical proparacaine hydrochloride 0.5% ophthalmic solution (Allergan plc) was administered before the measurement. All follow-up IOP measurements were taken at least 2 days after the implant injection procedure.

As an internal control for studies 3, 4, and 5, the IOP response of the animals to topical bimatoprost was tested before the study. One drop of bimatoprost 0.03% ophthalmic solution (Lumigan; Allergan plc) was administered in the morning once daily for 3 days in 1 eye (on study days −16, −15, and −14), followed by a washout of 2 weeks. IOP was measured in both eyes on day −16 before treatment and on days −15, −14, and −2 (baseline IOP assessment for the study).

IOP measurements at baseline, weeks 1, 2, and 3, and months 1, 1.5, and 2.5 or 3 were taken for both eyes in each study and were used for the pooled data analysis of average percentage change in IOP from baseline over 3 months.

## Results

### Study using topical bimatoprost

**FIG. 1.**
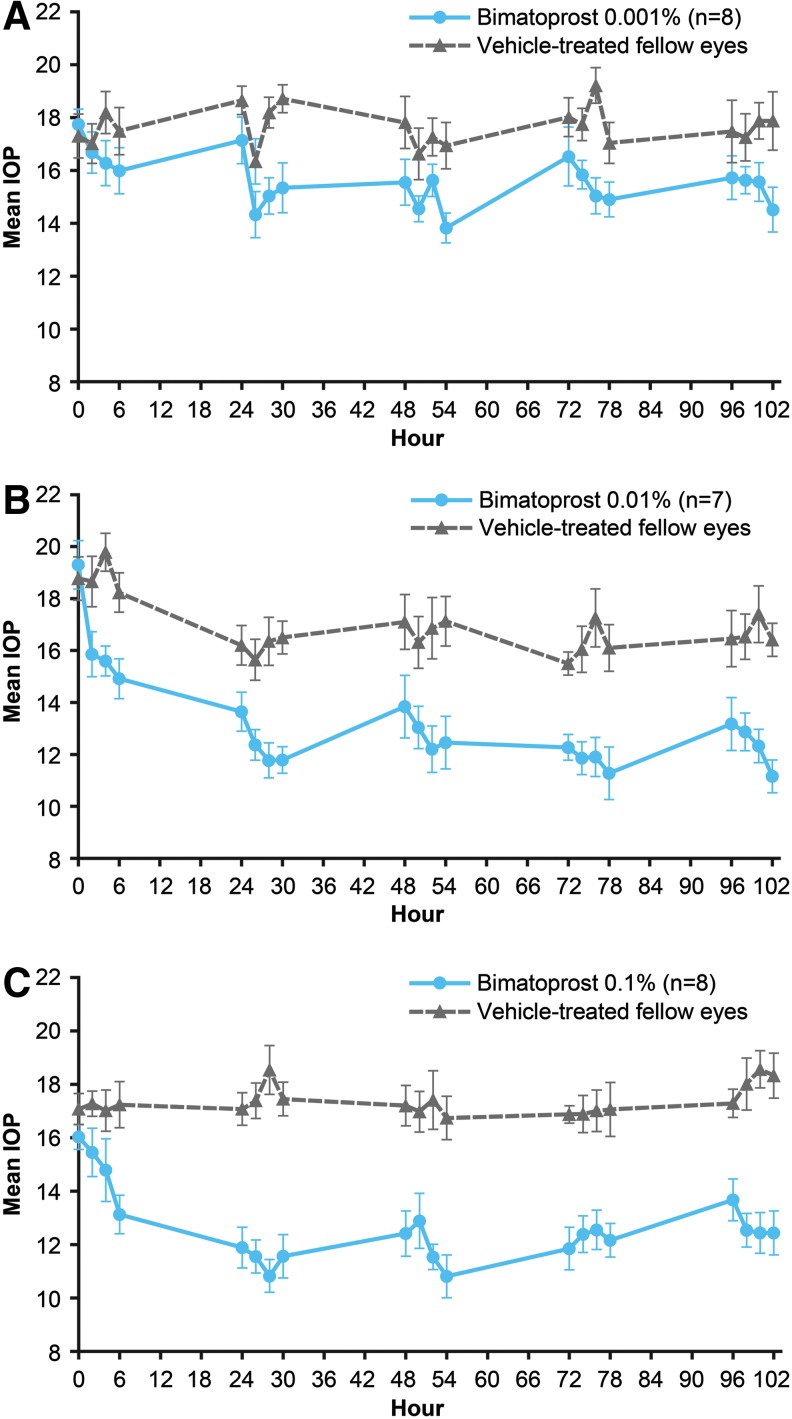
Mean IOP in the study eye of beagle dogs treated topically with **(A)** bimatoprost 0.001%, **(B)** bimatoprost 0.01%, or **(C)** bimatoprost 0.1% twice daily for 5 days. All fellow eyes received vehicle. Error bars indicate the standard error of the mean. IOP, intraocular pressure.

**FIG. 2.**
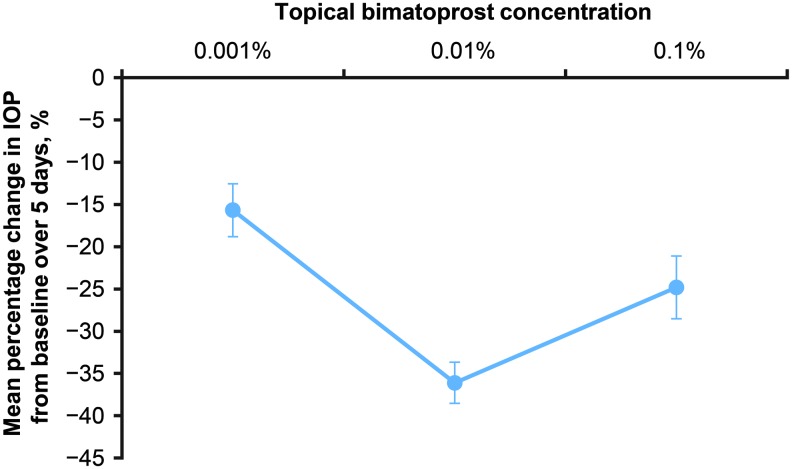
Mean peak (hour 6) percentage change in IOP from baseline in the study eye of beagle dogs over 5 days of twice-daily topical treatment with bimatoprost 0.001% (*n* = 8), bimatoprost 0.01% (*n* = 7), or bimatoprost 0.1% (*n* = 8). Baseline mean IOP in the study eye was 17.7, 19.3, and 16.0 mmHg and mean change from baseline IOP at hour 6 over 5 days was −2.8, −7.0, and −4.0 mmHg in the bimatoprost 0.001%, 0.01%, and 0.1% groups, respectively. Error bars indicate the standard error of the mean.

All 3 concentrations of topically applied bimatoprost lowered IOP in treated eyes (Fig. 1). The maximal decrease in IOP from the baseline IOP was generally at hour 6 (Fig. 1). At hour 6 on day 5, the mean IOP reduction from baseline was 3.2 mmHg (18.2%), 8.1 mmHg (42.2%), and 3.6 mmHg (22.4%) in eyes treated with bimatoprost 0.001%, 0.01%, and 0.1%, respectively. Averaging of the hour 6 mean percentage change in IOP from baseline across the 5 study days resulted in a U-shaped dose–response to topical bimatoprost (Fig. 2). Mean percentage IOP lowering increased from 15.7% to 36.1% as the bimatoprost concentration increased from 0.001% to 0.01%, but a decrease in IOP-lowering efficacy occurred as the bimatoprost concentration was further increased to 0.1%, and mean percentage IOP lowering decreased to 24.8% (Fig. 2).

### Studies using Bimatoprost SR

Five studies evaluated the IOP-lowering efficacy of 1 or more dose strengths of Bimatoprost SR. The dose strengths ranged from 8 to 120 μg of bimatoprost, with 3–8 animals used for each dose strength. In 3 of these studies, topical bimatoprost was administered in the prestudy period as an internal control. In the animals (*n* = 42) that received topical bimatoprost 0.03% once daily in the morning for 3 days, a mean peak IOP reduction of 5.9 mmHg (28.6%) from a baseline mean IOP of 20.6 mmHg was measured in the treated eye in the afternoon (6 h after dosing) on the third day. The mean percentage IOP reduction from baseline at hour 6 on the third day was 27.6%. Following cessation of the topical treatment and washout, the mean IOP in these animals was confirmed to be at pretreatment levels on study day −2 (baseline).

**FIG. 3.**
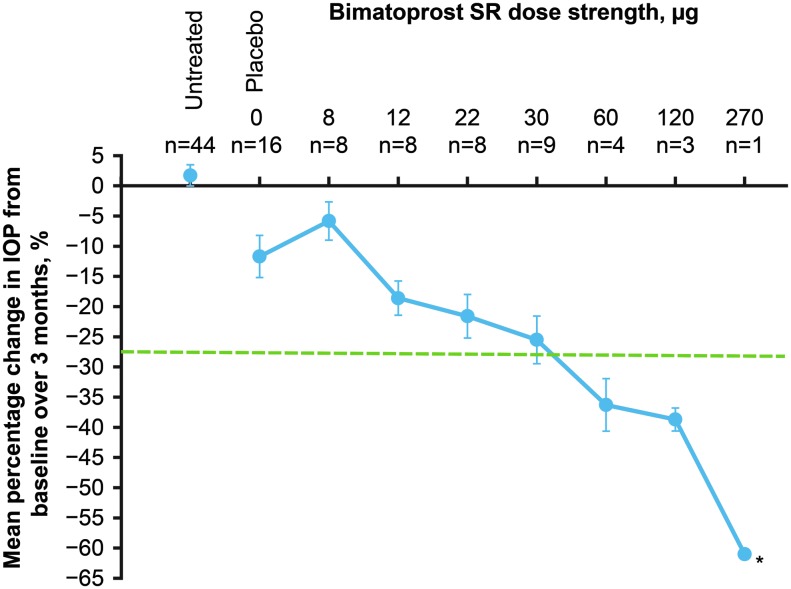
Mean percentage change in IOP from baseline in beagle dogs in the treated eye over 3 months after intracameral administration of Bimatoprost SR or a placebo implant. Error bars indicate standard error of the mean. *Dotted line* indicates the peak IOP reduction produced by 3 days of topical bimatoprost 0.03% treatment (*n* = 42). *Asterisk* indicates the sentinel dog data. Bimatoprost SR, bimatoprost sustained-release implant.

IOP lowering during the 3 months after administration of Bimatoprost SR was evaluated using the pooled study data. Table 2 shows mean IOP at baseline and during follow-up, as well as the mean change in IOP from baseline across follow-up measurements. Analysis of the percentage change in IOP from baseline showed that a decrease from baseline IOP greater than that observed in the placebo-treated eyes occurred with the Bimatoprost SR 12-μg dose and increased with increasing Bimatoprost SR dose strength throughout the range of dose strengths tested (Fig. 3). Furthermore, at the higher dose strengths of Bimatoprost SR (60 and 120 μg), the implant produced greater IOP reduction than topical bimatoprost 0.03% (Fig. 3).

**Table 2.** Intraocular Pressure in Bimatoprost Sustained-Release Implant Studies in Beagle Dogs

*Treatment*	*No. of eyes*	*Mean (SD) IOP, mmHg*							*Mean (SD) change in IOP from baseline over 3 months, mmHg [percentage change]*
		*BL*	*W1*	*W2*	*W3*	*M1*	*M1.5*	*M2.5 or M3*	
Untreated	44	19.7 (2.7)	19.5 (2.5)	19.7 (2.8)	19.8 (2.8)	20.2 (3.3)	20.6 (2.6)	19.0 (3.0)	+0.1 (2.3) [+0.7]
Placebo	16	19.7 (3.1)	15.7 (2.6)	16.6 (2.7)	17.6 (2.6)	18.4 (3.0)	18.5 (3.1)	15.9 (3.3)	−2.5 (3.2) [−13.0]
Bimatoprost SR 8 μg	8	18.4 (3.0)	15.8 (2.7)	15.7 (2.1)	17.7 (2.6)	17.9 (2.5)	17.8 (1.9)	17.9 (2.9)	−1.3 (1.8) [−6.8]
Bimatoprost SR 12 μg	8	19.4 (2.3)	14.1 (2.1)	16.1 (2.1)	15.1 (2.8)	16.4 (3.1)	17.2 (2.1)	15.2 (2.1)	−3.7 (1.8) [−19.1]
Bimatoprost SR 22 μg	8	19.8 (2.7)	14.6 (1.7)	15.6 (2.0)	15.4 (3.1)	16.4 (2.9)	16.7 (3.7)	13.7 (2.1)	−4.4 (2.4) [−22.2]
Bimatoprost SR 30 μg	9	21.6 (3.7)	14.5 (2.5)	15.4 (2.1)	16.1 (2.7)	17.6 (2.4)	16.4 (2.4)	15.0 (2.6)	−5.7 (3.2) [−26.8]
Bimatoprost SR 60 μg	4	22.0 (2.0)	14.0 (0.7)	15.0 (1.4)	15.4 (0.9)	13.1 (2.3)	14.1 (1.3)	11.8 (1.8)	−8.1 (2.7) [−36.8]
Bimatoprost SR 120 μg	3	18.7 (3.3)	11.5 (2.5)	11.3 (2.8)	12.0 (1.3)	11.7 (1.2)	11.2 (2.9)	11.0 (2.3)	−7.2 (1.4) [−38.7]

BL, baseline; M, month; W, week.

Miosis is a prominent side effect of topical PGAs in some species, including dogs.^13,14^ In these dog studies, all dose strengths of Bimatoprost SR were associated with miosis. Ocular hyperemia also occurred, but as reported in a previous study of Bimatoprost 30 μg in dogs,^15^ vasodilation was limited to aqueous outflow vessels, and the generalized dilation of the conjunctival and episcleral vasculature associated with topical bimatoprost administration was not observed.

## Discussion

In the studies reported here, the IOP-lowering efficacy of topical bimatoprost in normotensive beagle dogs was maximal at a bimatoprost concentration of 0.01%, and efficacy was reduced when the bimatoprost concentration was increased to 0.1%. In contrast, the IOP-lowering efficacy demonstrated by Bimatoprost SR consistently increased as the dose strength of Bimatoprost SR was increased. Furthermore, dose strengths of Bimatoprost SR above 30 μg produced greater IOP lowering than was produced by a maximally effective concentration of topically administered bimatoprost (0.03%).

In contrast to findings in human eyes, twice-daily dosing of topical PGA (latanoprost 0.005% or bimatoprost 0.03%) in beagle dogs has been shown to more effectively control IOP than once-daily dosing.^13,14^ Because of this species difference, which likely is explained by the large eye size and greater aqueous turnover in dogs, twice-daily dosing was used in the topical study. Increasing concentrations of topical bimatoprost dosed twice daily showed a ceiling effect on IOP lowering in dogs, consistent with results of human studies of topically administered bimatoprost and latanoprost, in which increasing the drug concentration or the dosing frequency of the topical PGA resulted in either no change or a decrease in IOP-lowering efficacy.^5,7–10^ In a concentration-ranging study of once-daily latanoprost in patients with glaucoma or ocular hypertension, the maximum effect on IOP was achieved with the 0.005% (marketed) concentration of latanoprost.^10^ Results in the per protocol patient population were analyzed for the worse eye using an analysis of covariance with baseline IOP as the covariate. The analysis showed that reductions from baseline IOP were numerically lower with higher concentrations of latanoprost, and at 8 am at week 4, latanoprost 0.005% reduced IOP by 1.1 mmHg (90% confidence interval, 0.3–1.8) more than latanoprost 0.0125%, the highest concentration tested, suggesting a U-shaped dose–response curve.^10^

It is unclear why increasing the concentration and dosing frequency of topical PGAs beyond an optimal level results in either no increase or a decrease in IOP-lowering efficacy. One possible explanation is the development of receptor subsensitivity.^7^ Other possible explanations include mixed agonist/antagonist effects (ie, PGA functions as agonist and antagonist at different receptor types that when activated, result in IOP reduction), and activation of multiple receptor types, with different affinity, that have competing effects on IOP. In addition, it has been suggested that saturation of either PGA uptake or PGA metabolism to active drug (for PGA prodrugs) might be involved.^10^ Saturation of PGA uptake or metabolism could account for a plateau in effect, but how it might lead to diminished effect with increased drug exposure is not readily apparent.

The normotensive beagle dog is a useful model for evaluation of the clinical potential of IOP-lowering medications.^16–18^ The larger iridocorneal angle in the dog eye, however, can fit a larger implant than is possible in the human eye. Consequently, a larger range of Bimatoprost SR dose strengths can be evaluated in dog studies than in clinical studies.

In the phase 1/2 clinical study of Bimatoprost SR, no ceiling effect in IOP lowering was observed with increasing implant dose strength, but the highest dose strength of implant evaluated was 20 μg,^11^ because this was the largest initial dose that could fit in the iridocorneal angle. A trend for a dose–response was observed in the first 16 weeks postinjection, with average IOP reductions over 16 weeks of 7.2, 7.4, 8.1, and 9.5 mmHg with the 6-, 10-, 15-, and 20-μg dose strengths of Bimatoprost SR, respectively.^11^ Eyes treated with the 20-μg dose strength showed a larger IOP reduction compared with control eyes treated with 0.03% bimatoprost; the mean decrease in IOP from baseline over 16 weeks was 9.5 and 8.4 mmHg with Bimatoprost SR 20 μg and bimatoprost 0.03%, respectively.^11^ A dose strength of Bimatoprost SR higher than 20 μg might have provided larger reductions in IOP.

Our studies of Bimatoprost SR in normotensive beagle dogs evaluated dose strengths up to 120 μg, and a single additional animal received a dose strength of 270 μg. In contrast to the U-shaped dose–response curve observed with topical bimatoprost dosing, increasing dose strengths of Bimatoprost SR provided additional IOP lowering throughout the range of dose strengths tested, and the higher dose strengths tested produced greater IOP reductions than were achieved with topical dosing. These results suggest that in the future, it may be possible to reformulate a bimatoprost implant with a higher dose, or use a more potent prostamide in place of bimatoprost, and develop an implant that can lower IOP more effectively than both topical administration and the current generation of Bimatoprost SR.

It is possible that a high-dose strength of Bimatoprost SR can provide greater IOP lowering than topical bimatoprost administration in dogs because of its effects on episcleral venous pressure (EVP), an important determinant of IOP.^19^ A study in normotensive beagle dogs showed that administration of the 30-μg implant causes selective dilation of aqueous outflow vessels, unlike topical bimatoprost administration, which causes generalized ocular surface vasodilation.^15^ Differences in effects of Bimatoprost SR and topical bimatoprost on the vasculature presumably result from differences in drug localization after intracameral and topical drug delivery. The selective dilation of aqueous outflow vessels produced by Bimatoprost SR was associated with a sustained decrease in EVP after an initial transient increase,^15^ and this sustained decrease in EVP can be expected to result in additional IOP reduction.

No currently available IOP-lowering medications have been shown to reduce EVP. However, the mechanism of action of topical netarsudil, a Rho kinase inhibitor in development for IOP lowering in glaucoma, has been reported to involve a decrease in EVP as well as an increase in trabecular outflow facility.^20,21^ Bimatoprost SR may lower IOP through a mechanism involving reduction of EVP, as well as increases in uveoscleral and trabecular outflow, but studies of the effects of Bimatoprost SR on aqueous dynamics are needed to fully elucidate its mechanism of IOP lowering.

The additional IOP reduction with Bimatoprost SR might also be explained by the levels of bimatoprost achieved in target tissues after implant administration. The maximal bimatoprost concentration in the iris-ciliary body (a target tissue for lowering IOP) in beagle dogs was 4 log units higher after Bimatoprost SR 15 μg administration than after 7 days of once-daily topical dosing with bimatoprost 0.03%.^22^ It is possible that this very high concentration of bimatoprost might cause anatomic or physiological changes in the target tissue that have additional effects on IOP. No histopathology of the ciliary muscle was done in this study. However, topical dosing of bimatoprost in cynomolgus monkeys over 12 months was shown to lead to remodeling of the anterior ciliary muscle with enlarged outflow channels incompletely lined with elongated endothelial cell–like cells.^23^ Changes in the extracellular matrix were also observed in the juxtacanalicular trabecular meshwork.^23^ This remodeling may be the consequence of a stimulation of matrix metalloprotease synthesis by ciliary muscle cells in response to treatment with a PGA.^24^ With Bimatoprost SR, sustained drug release and higher drug concentrations in the ciliary body may lead to more durable remodeling changes, which may translate to greater duration of IOP reduction. Such changes could explain the longevity of the effects of Bimatoprost SR in patients with glaucoma. In the phase 1/2 trial of Bimatoprost SR, at study completion 2 years after a single administration of implant, 28% of patients still had not required topical IOP-lowering rescue medication or implant retreatment.^25^

A limitation of this work is that the studies of topical treatment and Bimatoprost SR were conducted at different times. However, both studies were designed and overseen by Allergan, and all data analysis was performed at Allergan. Comparisons of results between the topical treatment and implant studies are facilitated by similarities between the studies, including use of research normotensive, purebred beagle dogs in both studies, and measurement of IOP using validated pneumatonometry in nonsedated animals in both studies.

In summary, we have demonstrated that in normotensive beagle dogs, the IOP-lowering response to topical bimatoprost is limited at high concentrations of bimatoprost, whereas the IOP lowering provided by Bimatoprost SR is related to the dose strength of the implant, with no plateau in effect at dose strengths up to 270 μg. At dose strengths of 60 μg and higher, the IOP lowering produced by Bimatoprost SR surpassed that produced by topical bimatoprost administration. The maximum achievable IOP lowering with Bimatoprost SR in dogs is not yet known. Additional studies are needed to explore the mechanisms for the additional IOP lowering provided by Bimatoprost SR in dogs and to determine the extent to which these preclinical findings translate to humans.
